# Long‐term results of the FIL MCL0208 trial of lenalidomide maintenance versus observation after ASCT in MCL patients

**DOI:** 10.1002/hem3.70102

**Published:** 2025-03-22

**Authors:** Rita Tavarozzi, Simone Ferrero, Andrea Evangelista, Elisa Genuardi, Daniela Drandi, Michael Mian, Manuela Zanni, Federica Cavallo, Alice Di Rocco, Vittorio Stefoni, Chiara Pagani, Alessandro Re, Barbara Botto, Monica Balzarotti, Vittorio R. Zilioli, Maria Gomes da Silva, Luca Arcaini, Anna L. Molinari, Filippo Ballerini, Andrés J. M. Ferreri, Benedetta Puccini, Carlo Visco, Piero M. Stefani, Mario Luppi, Ivana Casaroli, Caterina Stelitano, Giovannino Ciccone, Umberto Vitolo, Maurizio Martelli, Sergio Cortelazzo, Marco Ladetto

**Affiliations:** ^1^ Department of Translational Medicine University of Eastern Piedmont Novara Italy; ^2^ SCDU of Hematology, AOU SS. Antonio e Biagio e Cesare Arrigo Alessandria Italy; ^3^ Department of Molecular Biotechnologies and Health Sciences University of Torino Turin Italy; ^4^ AOU Città della Salute e della Scienza di Torino Turin Italy; ^5^ Unit of Cancer Epidemiology, CPO, AOU Città della Salute e della Scienza di Torino Turin Italy; ^6^ Department of Hematology and Center of Bone Marrow Transplantation Hospital of Bolzano Bolzano Italy; ^7^ Hematology Department of Translational and Precision Medicine Sapienza University of Rome Rome Italy; ^8^ Institute of Hematology “L. e A. Seràgnoli” University of Bologna Bologna Italy; ^9^ UOC Ematologia, Spedali Civili di Brescia Brescia Italy; ^10^ Hematology Unit IRCCS Humanitas Research Hospital Rozzano Milan Italy; ^11^ Division of Hematology ASST Grande Ospedale Metropolitano Niguarda Milan Italy; ^12^ Departamento de Hematologia Instituto Portugues de Oncologia de Lisboa Lisbon Portugal; ^13^ Department of Molecular Medicine University of Pavia Pavia Italy; ^14^ Division of Hematology Fondazione IRCCS Policlinico San Matteo Pavia Italy; ^15^ Unità Operativa di Ematologia Ospedale degli Infermi di Rimini Rimini Italy; ^16^ IRCCS‐Ospedale Policlinico San Martino Genoa Italy; ^17^ Unità Linfomi, IRCCS Ospedale San Raffaele Milan Italy; ^18^ SOD Ematologia AOU Careggi Florence Italy; ^19^ Department of Medicine Section of Hematology University of Verona Verona Italy; ^20^ Hematology Unit, General Hospital Treviso Treviso Italy; ^21^ Department of Medical and Surgical Sciences UNIMORE Modena Italy; ^22^ IRCCS San Gerardo Monza Italy; ^23^ Azienda Ospedaliera Bianchi‐Melacrino‐Morelli Reggio Calabria Italy; ^24^ Fondazione del Piemonte per l'Oncologia‐IRCCS, Candiolo Cancer Institute Candiolo Italy; ^25^ Ospedale Centrale di Bolzano Bolzano Italy

Mantle cell lymphoma (MCL) is an uncommon subtype of B‐cell non‐Hodgkin lymphoma (NHL) not easily manageable due to chemotherapy resistance and tendency to relapse.^1^ Current treatment for young, fit patients with MCL consists of induction treatment with rituximab and ARA‐C‐based chemotherapy, followed by consolidation with autologous stem cell transplantation (ASCT) and immunotherapy maintenance.^2^, ^3^, ^4^ More recently, the potential value of immunomodulatory agents and Bruton tyrosine kinase inhibitors during induction and maintenance has been investigated, and the role of consolidation ASCT is now debated.^5^, ^6^, ^7^

The FIL MCL0208 (clinicaltrials.gov no. 02354313) phase III trial evaluated the efficacy of lenalidomide maintenance (LEN) versus observation (OBS) after ASCT in younger, fit patients (18–65 years) with untreated advanced‐stage MCL. Further details are provided in Supporting Information. The trial enrolled 303 patients across 38 centers (37 in Italy, 1 in Portugal). At the primary endpoint analysis, with a median follow‐up of 38 months, LEN demonstrated a progression‐free survival (PFS) benefit but no overall survival (OS) advantage.^6^ Minimal residual disease (MRD) monitoring in peripheral blood (PB) and bone marrow (BM), conducted via nested and real‐time PCR (RQ‐PCR) at 10 predefined time points, highlighted MRD's prognostic value for time to progression (TTP).^8^

This report presents long‐term clinical and molecular outcomes with a median follow‐up of 74 months, including four additional late MRD assessments at 18, 24, 30, and 36 months, expanding insights into LEN's impact and the prognostic role of MRD over time.

The sample size determination and the statistical plan analyses were described previously.^6^ Further details are provided in Supporting Information.^9^, ^10^

From May 4, 2010 to August 24, 2015, a total of 303 patients entered the study. Clinical characteristics at enrollment were published previously.^6^ The PFS and OS of both the enrolled and randomized populations are described in the original report.^6^

At the time of the present long‐term analysis, the median follow‐up was 84 months from enrollment and 73 months for the randomized population. The median PFS of the enrolled population was 64 (95% confidence interval [CI] 56–85) months, and the median OS of the enrolled population was not reached. The 72‐month PFS was 48% (95% CI 42–54) and the 72‐month OS 75% (95% CI 70–80). At the time of the present long‐term analysis, 44 of 104 patients in the LEN arm had a PFS event compared to 51 of 101 patients in the OBS arm. The median PFS from randomization was 76 (95% CI 56‐not reached) months in the LEN arm versus 73 (95% CI 46–90) months in the OBS arm. The 72‐month PFS rates were 55% (95% CI 44–65) and 50% (95% CI 39–60), respectively. This resulted in a stratified HR of 0.76 (95% CI 0.51–1.14) with a log‐rank test *p*‐value of 0.177 (Figure 1A). The median OS from randomization was not reached in either arm. At 72 months, the OS rates were very close between the LEN (77%, 95% CI 66–85) and OBS arms (75%, 95% CI 64–83), with a stratified HR of 0.94 (95% CI 0.53–1.66) and *p* = 0.828 in the log‐rank test (Figure 1B).

**Figure 1:**
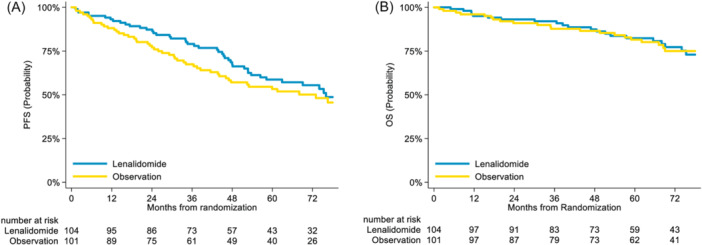
**Survival analysis. (A)** Progression‐free survival (PFS) of the randomized population. **(B)** Overall survival (OS) of the randomized population.

The landmark analysis showed a PFS advantage in favor of LEN up to 36 and 42 months (HR 0.58 [95% CI 0.33–1.00], *p* = 0.048 and HR 0.57 [0.34–0.95], *p* = 0.032, respectively; Figures 2A,B). However, a subsequent decrease in PFS benefit was observed during the follow‐up period after 36 and 42 months (HR 1.07 [0.59–1.93], *P* = 0.83 and HR 1.21 [0.63–2.32], *P* = 0.565; Figures 2C,D). In the LEN arm, the monthly HR for PFS during the first 24 months postrandomization (when LEN was administered) ranged from 0.5% to 0.7%. This rate gradually narrowed (0.7%–0.95%) from 24 to 36 months before eventually becoming comparable between the two arms (~1% from 36 to 60 months; Figure 2E). The flexible parametric survival model also suggests that LEN provides an initial benefit over OBS, with a significantly lower HR in the early months, gradually approaching 1 during long‐term follow‐up (Figure 2E). We also conducted a subgroup analysis of PFS to evaluate whether the effect of LEN was more pronounced in specific subgroups of patients. Consistent with the results of the earlier midterm analysis, patients with no BM involvement at diagnosis seemed to benefit more from the experimental treatment (HR 0.24 vs. 1.09, *p* = 0.004). Similarly, among patients with CR and a PCR‐negative response, a more pronounced benefit from LEN was observed compared to other patients, though with weaker evidence (HR 0.52 vs. 0.97, interaction *p* = 0.150). An additional subgroup analysis not included in the main manuscript showed a significantly larger efficacy on blastoid histology compared to classical histology (HR 0.11 vs. 0.80, *p* = 0.007). Other investigated parameters (e.g., MIPI systemic symptoms, bulky disease, Ki‐67, and TP53 mut/del) did not seem to have an impact on the effect of LEN (Supporting Information).

**Figure 2:**
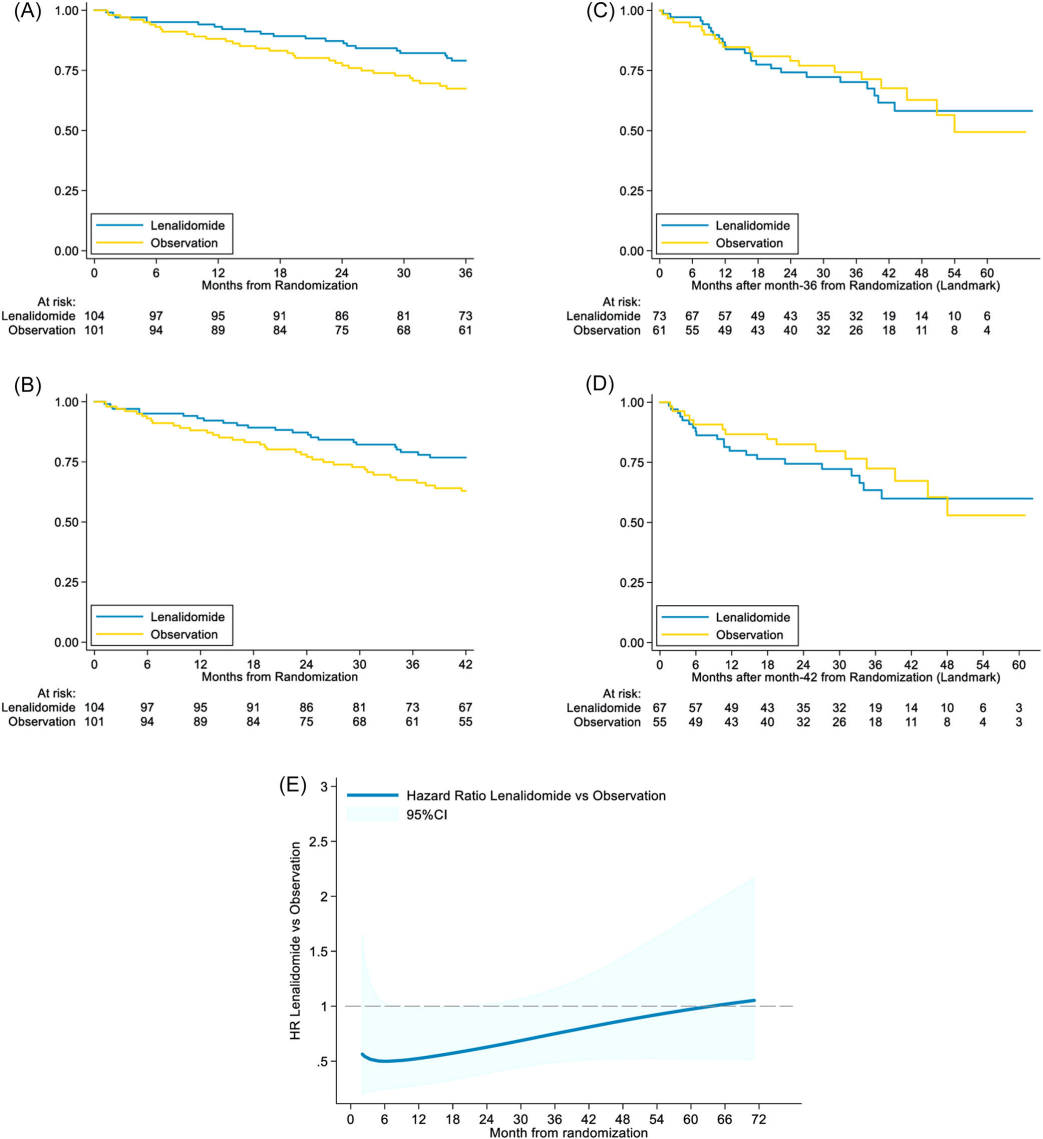
**Landmark analysis. (A, B)** Up to 36 and 42 months after randomization. **(C, D)** After 36 and 42 months after randomization. **(E)** Time‐dependent hazard ratio (HR) of lenalidomide maintenance versus observation with 95% confidence interval (Royston‐Parmar model).

Globally, 28 secondary malignancies occurred in the enrolled population (60‐month incidence from consent 8.0% [95% CI 5.2–11.6]). Twelve secondary malignancies occurred in the nonrandomized population (60‐month incidence from consent 10.4% [3.9–16.9]). Sixteen secondary malignancies occurred in the randomized population (60‐month incidence from randomization 6.9% [3.1–10.6]). We recorded 10 (4.5%) solid tumors in the randomized population: 7 (6%) in the LEN arm (one stomach, one prostate, one gallbladder, one pancreas, one bladder, one lung, and one skin) and 3 (3%) in the OBS arm (one lung, one bowel, and one prostate). Five (3%) cases of secondary myelodysplastic syndrome (MDS) and acute myeloid leukemia (AML) were recorded in the randomized population: 3 (3%) in the LEN arm and 2 (1%) in the OBS arm. Including only patients in the LEN safety population or randomized to OBS, the cumulative incidence of any secondary malignancy at 60 months was 9.4% (3.1–15.6) in the LEN safety population versus 4.5% (0.1–8.8) in the OBS arm (MIPI‐adjusted HR 1.98 [95% CI 0.67–5.87], *p* = 0.219; Supporting Information).

The report on MRD early detection time points was published previously.^8^ Here, we provide long‐term molecular results for two additional late MRD time points (30 and 36 months, see the Supporting Information), as well as an updated survival analysis including OS. Overall, using time‐varying covariates and adjusting the HR for MIPI, MRD positivity at any time point conferred a global HR for TTP of 4.17 if detected in BM (95% CI 2.70–6.44, *p* < 0.001) or 2.64 if detected in PB (95% CI 1.69–4.12, *p* < 0.001). Moreover, the global HR for OS was 2 for MRD positivity detected in BM (95% CI 1.13–3.53, *p* = 0.017) and 1.60 in PB (95% CI 0.87–2.95, *p* = 0.13; Supporting Information). In landmark analysis, MRD positivity in any tissue at any given time point during the 3 years after ASCT continued to predict dismal TTP (Supporting Information). No significant difference was noted in the impact of MRD according to randomization arm, as reported previously (data not shown).^8^

The MRD monitoring over time (“MRD kinetics”) predicted patients' outcomes better than a single time point. The accumulation of two or more negative results for MRD during the 3 years after ASCT predicted a progressively more favorable TTP (HR 0.40–0.13 in BM and HR 0.65 to >0.19 in PB) and OS (HR 0.47–0.35 in BM and HR 0.96 to >0.42 in PB), as described in the Supporting Information. Similarly, the calculation of time‐varying AUCs confirmed that the predictive model combining MRD kinetic evaluation and MIPI (Supporting Information) outperformed, in terms of TTP, the composite model based on MIPI and MRD single time point analysis (see Supporting Information) when MRD was measured in both BM and PB samples.

The long‐term analysis of the FIL MCL0208 trial confirms that the R‐HDS^11^, ^12^, ^13^ regimen is feasible and provides comparable results to other immunochemotherapy regimens in similar populations. However, it was noted to be more cumbersome and toxic than other ASCT‐containing regimens. In this trial, LEN showed significant short‐term PFS benefits during the 2 years of active treatment. Unfortunately, this benefit was not maintained long term, as PFS events increased after LEN discontinuation, and there was no difference in OS. Comparatively, the LYMA study^2^ demonstrated more sustained PFS benefits with rituximab maintenance, and the MCL‐R2^14^ trial suggested greater efficacy for LEN while combined with rituximab rather than as a single agent.

This trial might arise a concern regarding secondary malignancies, with a 7‐year cumulative incidence of 9.4% in the LEN arm compared to 4.5% in the observation arm. Although these differences were not statistically significant, they align with existing literature, emphasizing the need for prolonged patient monitoring.

The MRD substudy reinforced the prognostic value of MRD, particularly kinetic models, in predicting progression. PB was validated as a reliable source for MRD monitoring over time. These findings suggest that MRD‐negative patients might benefit from treatment deintensification, warranting exploration in future trials.

Recent advances, including the European MCL Network's TRIANGLE trial, highlight a paradigm shift in MCL treatment.^5^, ^15^, ^16^ The addition of ibrutinib during induction and as maintenance, with or without ASCT, showed significant efficacy. This underscores the importance of selecting optimal maintenance therapies or combinations, especially as treatment strategies move toward less intensive chemotherapy and reduced reliance on ASCT in younger MCL patients.

## AUTHOR CONTRIBUTIONS

Marco Ladetto, Sergio Cortelazzo, Umberto Vitolo, and Giovannino Ciccone designed the research study. Marco Ladetto, Giovannino Ciccone, Andrea Evangelista, Simone Ferrero, and Rita Tavarozzi designed and completed the database. Andrea Evangelista and Giovannino Ciccone conducted the statistical analysis. Marco Ladetto, Rita Tavarozzi, Simone Ferrero, and Andrea Evangelista were involved in data analysis and interpretation. Rita Tavarozzi, Marco Ladetto, Simone Ferrero, Andrea Evangelista, and Giovannino Ciccone wrote the manuscript. Elisa Genuardi performed MRD molecular analysis, and all authors reviewed and approved the manuscript.

## CONFLICT OF INTEREST STATEMENT

Rita Tavarozzi: speaker's honoraria from Lilly, SOBI. Simone Ferrero: consultant for Janssen, EUSA Pharma, AbbVie, and Sandoz; is on the advisory board of Janssen, EUSA Pharma, Recordati, Incyte, Roche, AstraZeneca, and Italfarmaco; received speaker's honoraria from Janssen, EUSA Pharma, Recordati, Lilly, BeiGene, Gilead, and Gentili; and received research funding from Gilead, BeiGene, and Morphosys. Alice Di Rocco: advisory board: Incyte and Takeda; invited speaker: Takeda and SOBI. Maria Gomes da Silva: research grants: Gilead Sciences and AstraZeneca; advisory boards: Janssen, Roche, Gilead Sciences, Lilly, and Takeda; institutional payments: Janssen and AbbVie. Vittorio R. Zilioli: advisory boards: Kite/Gilead and Takeda; consultancy: Roche; research funding: Kite/Gilead; speakers bureau: Janssen, Lilly, and Takeda; other (travel expenses): BeiGene, Janssen, Roche, and Takeda. Monica Balzarotti: advisory board: Roche, Gilead, Eli Lilly, AstraZeneca, and AbbVie/GenMab; speaker's bureau: Roche, Gilead, Eli Lilly, SOBI, and BeiGene; Andrés J. M. Ferreri: advisory board, personal: AbbVie, AstraZeneca, BMS, Genmab, Gilead, Incyte, Juno, Novartis, PletixaPharm, Roche, Serb, and SOBI; BTG Therapeutics, research grant, institutional, no financial interest; Serb, research grant, institutional, no financial interest; Takeda, local PI, institutional, no financial interest. Federica Cavallo: speaker honoraria from Lilly, Incyte, Roche, BeiGene, Servier, Novartis, and Gilead; advisory boards for Roche and Incyte; consultant for AstraZeneca. Chiara Pagani: advisory board/consultation: Takeda and Sandoz. Luca Arcaini: honoraria: EUSA Pharma and Novartis. Advisory boards/consultation: Roche, Incyte, EUSA Pharma, Kite/Gilead, Novartis, and Morphosys. Piero M. Stefani: advisory honoraria and travel expenses from Takeda, Roche, Janssen‐Cilag, EUSA Pharma, and Incyte. Mario Luppi: advisory board and meeting with honoraria: AbbVie, Jazz Pharma, Novartis, Grifols, Sanofi, Incyte, Istituto Gentili, Roche, and AstraZeneca. Michael Mian: advisory boards: Janssen, Roche, Gilead Lilly, Takeda, Novartis, BMS, AstraZeneca, BeiGene, Incyte, and Recordati; speaker bureau: Roche and Gilead. Mario Luppi: consultancy, participation to advisory boards, invitation to scientific meetings, institutional research support and contracts with AbbVie, Acerta, Amgen, ADC Therapeutics, BeiGene, Celgene/BMS, EUSA Pharma, GSKI, Gentili, Gilead/Kite, Novartis, Incyte J&J, Jazz, Lilly, Regeneron, Roche, and Sandoz.

### FUNDING

This work was supported by Fondazione Italiana Linfomi and Celgene. Further support derived from the grant number MCL 7005‐24 of Leukemia and Lymphoma Society (LLS), Progetto di Ricerca Sanitaria Finalizzata 2021 (RF‐2021‐12371972) and AIL AL‐ASTI ODV.

## Supporting information

Supporting information.

## Data Availability

The data that support the findings of this study are available from the corresponding author upon reasonable request.
