# Supplementary material for: Long‐term results of the FIL MCL0208 trial of lenalidomide maintenance versus observation after ASCT in MCL patients
Source: Hemasphere. 2025 Mar 22;9(3):e70102. doi: 10.1002/hem3.70102 (PMC11928766; doi:10.1002/hem3.70102)

*Study design and participants*

FIL-MCL0208 (NCT02354313) is a phase 3, multicenter, open-label, randomized, controlled trial, designed to evaluate the efficacy of LEN versus OBS after ASCT in younger (18-65 years old), fit, advanced stage (Ann Arbor III-IV or stage II plus bulky disease [≥5 cm] or B symptoms) previously untreated MCL patients. The study involved 38 FIL centers. Further details on inclusion/exclusion criteria and study design are provided elsewhere. ^6^

This unblinded study was conducted according to the principles of the Declaration of Helsinki and in adherence with Good Clinical Practice Standards. The protocol was reviewed and approved by the independent ethics committee of all participating centers. Written informed consent (WIC) was obtained from all patients. All patients also provided written informed consent for the use of their biological samples for research purposes, in accordance with Institutional Review Boards requirements and the Helsinki declaration.

This study is registered with EudraCT (2009–012807–25) and ClinicalTrials.gov (NCT02354313).

*Procedures*

Induction (HDS) and ASCT programs have been previously described, including treatment schedules, restaging procedures, prophylactic measures, and toxicity management in the first report of the trial. ^6^ After first line intensified R-high dose schedules (HDS) followed by autologous stem cell transplantation (ASCT), patients achieving complete (CR) or partial remission (PR) assessed by CT (CR or PR according the 2007 International Working Group Criteria 9 for non-PET avid lymphomas) and achieving hematological recovery (absolute neutrophil count [ANC] > 1·5×10^9^/L and platelets >60×10^9^/L) within 120 days after ASCT were randomized to receive LEN (15 mg daily for platelets >100×10^9^/L or 10 mg daily for platelets 60-100×10^9^/L, days 1-21 every 28 days) for 24 months or to undergo OBS. Treatment was continued for 24 months until disease progression or unacceptable toxicity or voluntary withdrawal of the patient. If any toxicity occurred, the dose of lenalidomide for each patient was interrupted or modified as previously described.^6^ The frequency and severity of adverse events observed were classified and tabulated based on National Cancer Institute Common Terminology Criteria for Adverse Events (CTCAE) Version 4.0. Prophylactic use of G-CSF was allowed and used to support ANC recovery and to prevent febrile neutropenia even when not specifically recommended. During maintenance/observation, patients underwent monthly physical examinations, ECOG-PS determination, complete blood counts and blood chemistry, and periodic quality of life assessments.

During follow-up, patients were evaluated with clinical evaluation, CT scan, laboratory tests at least every 3 months for the first year after end of treatment, and then every 6 months for 2 more years. After the third year, patients were followed up with visits according to the choice of the local hematologist. It was demanded that patients were seen at least 2 times per year for clinical examination and blood tests.

CT-scan was mandatory for any clinical suspicion of relapse or appearance of adenopathies or organomegaly or suspicious alterations of the blood exams. Queries were sent to the centers twice a year by FIL offices to update the state of disease and inform on late toxicities or secondary malignancies (SM).

*Outcomes*

The primary endpoint of the trial was PFS measured from the date of randomization to progression, relapse, or death from any cause, according to the 2007 Revised Criteria for Malignant Lymphoma for non-PET avid disorders. 9 Secondary endpoints were OS, Event-free survival (EFS), Disease-free survival (DFS), overall response rate (ORR), complete response rate (CRR), safety, secondary malignancies (SMs) and MRD related endpoints. Additional secondary endpoints were those based on quality of life and cost-effectiveness, which are not described in the present report. The primary end-point analyses have been detailed in the original paper. ^6^

We here provide long-term clinical and molecular results.

Toxicity was analyzed in terms of secondary malignancies reported by investigators during the follow-up period.

*MRD monitoring*

A systematic MRD monitoring plan by ASO RQ-PCR on immunoglobulin heavy chain (IGH) and *BCL1::IGH* clonal rearrangements was predefined from the start of the trial: study design and biological samples collection have been published elsewhere.6,8 All bone marrow (BM) aspirates and peripheral blood (PB) samples were centralized at baseline, during treatment and up to 36 months (M36) after randomization in the EuroMRD standardized laboratory of the University of Torino (Torino, Italy) (https://www.euromrd.org/usr/pub/pub.php).

*Statistical analysis*

Sample size determination, based on PFS after randomization, has been previously described. ^6^ Statistical analyses were performed according to different populations: the enrolled population (comprising all patients initially enrolled in the study), the ITT population (comprising all patients randomly assigned to either LEN or OBS), and the safety population (comprising all patients who received at least one dose of the assigned treatment). Time-to-event endpoints, including PFS, OS, EFS, and DFS, were estimated using the Kaplan-Meier method starting from the date of enrollment for the enrolled population and from the date of randomization for the ITT population. Differences between arms were evaluated using a stratified log-rank test, with strata defined according to clinical and molecular response at randomization. Hazard ratios (HRs) were estimated with the stratified Cox model and adjusting for the MIPI in an additional sensitivity analysis. To account for a potential change in LEN effect on PFS over time, we conducted additional analyses. In the first analysis, we included a time-dependent covariate in the Cox model. This covariate was employed to model the effect of LEN based on time elapsed since randomization, distinguishing between periods before and after 36 or 42 months. In the second analysis, we used a flexible parametric survival method ^9^ to model the PFS hazards of randomization arms as a function of time since randomization. These approaches enabled us to effectively address time-varying effects of treatment on PFS. The cumulative incidence of secondary malignancies was estimated using the method proposed by Gooley et al. ^10^ Death without secondary malignancy was considered as a competing event, and group comparisons were performed using the Fine and Gray model, adjusted for MIPI.

The prognostic role of MRD analysis was investigated concerning OS and TTP (time to progression, measured from the time of each MRD determination until lymphoma progression or death). Given the multiple updates of MRD status during follow-up, the effect of MRD positivity on OS and TTP was evaluated through various landmark analyses at specific time points (R-CTX, R-HD-ARAC, post-ASCT, M6, M12, M18, M24, M30, M36). A comprehensive analysis across the entire follow-up, using MRD positivity as a time-dependent variable, was conducted to avoid immortal time bias. This involved splitting patient follow-up time based on MRD determination dates and updating MRD status accordingly. The impact of MRD positivity on OS and TTP was estimated using the Cox model, adjusted for the MIPI score. MRD determinations within 60 days of the last follow-up for TTP were excluded to avoid overestimating the predictive ability regarding disease progression.

The discrimination ability of Cox models incorporating MRD determinations as a time-dependent variable was compared with models based on a single MRD assessment post-ASCT and post-M6, adjusting for MIPI and measuring OS and TTP from the M6 determination. Time-varying AUCs were estimated over follow-up time to evaluate model performance, using the risksetAUC function in R (package risksetROC).

Supplemental table 1 - Multivariate analysis on PFS

|  | **Univariable** | | **Multivariable** | |
| --- | --- | --- | --- | --- |
|  | HR | p | HR | p |
| MIPI Interm vs Low | 1.63 | 0.006 | 1.39 | 0.078 |
| MIPI High vs Low | 2.69 | <0.001 | 1.86 | 0.008 |
| Male vs Female | 1.81 | 0.008 | 1.82 | 0.008 |
| B symptomes | 1.52 | 0.014 | 1.19 | 0.344 |
| Bulky disease | 1.94 | <0.001 | 1.57 | 0.008 |
| Positive Bone | 1.58 | 0.030 | 1.27 | 0.272 |
| Ki67 High | 1.64 | 0.004 | 1.36 | 0.090 |
| MIPIC L-I vs L* | 1.15 | 0.482 | 1.17 | 0.435 |
| MIPIC H-I vs L* | 1.45 | 0.134 | 1.05 | 0.865 |
| MIPIC H vs L* | 2.82 | <0.001 | 2.22 | 0.004 |

Supplemental table 2 – Additional MRD timepoints

| **MRD Timepoint** | **Expected samples** | **Available MRD results (BM)** | **Compliance** | **Available MRD results (PB)** | **Compliance** |
| --- | --- | --- | --- | --- | --- |
| M30 | 142 | 45 | 32% | 44 | 31% |
| M36 | 140 | 43 | 31% | 40 | 29% |

Supplemental table 3 - Effect of MRD positivity on TTP and OS during follow-up using time-varying covariates. HRs were MIPI adjusted.

|  | BM | | | PB | | |
| --- | --- | --- | --- | --- | --- | --- |
|  | HR | 95% CI | p | HR | 95% CI | p |
| **MRD+ any time** |  |  |  |  |  |  |
| TTP | 4.17 | 2.70,6.44 | <0.001 | 2.64 | 1.69,4.12 | <0.001 |
| OS | 2.00 | 1.13,3.53 | 0.017 | 1.60 | 0.87,2.95 | 0.130 |

Supplemental table 4- TTP analysis according to cumulative negativity results.

| **TTP** | **BM HR** | **BM 95%CI** | **p** | **PB HR** | **PB 95%CI** | **p** |
| --- | --- | --- | --- | --- | --- | --- |
| Positive (ref) | 1 | - | - | 1 | - | - |
| 1 cumulative negativity | 0.50 | 0.27,0.95 | 0.036 | 0.89 | 0.48,1.64 | 0.711 |
| 2 cumulative negativities | 0.40 | 0.21,0.78 | 0.007 | 0.65 | 0.36,1.16 | 0.144 |
| 3 or more negativities | 0.13 | 0.07,0.24 | <0.001 | 0.19 | 0.10,0.34 | 0.000 |

Supplemental table 5- OS analysis according to cumulative negativity results.

| **TTP** | **BM HR** | **BM 95%CI** | **p** | **PB HR** | **PB 95%CI** | **p** |
| --- | --- | --- | --- | --- | --- | --- |
| Positive (ref) | 1 | - | - | 1 | - | - |
| 1 cumulative negativity | 1.13 | 0.51, 2.50 | 0.766 | 0.74 | 0.31, 1.76 | 0.493 |
| 2 cumulative negativities | 0.47 | 0.17, 1.25 | 0.129 | 0.96 | 0.45, 2.02 | 0.909 |
| 3 or more negativities | 0.35 | 0.16, 0.73 | 0.005 | 0.42 | 0.19, 0.90 | 0.025 |

Supplemental figure 1 – PFS of the enrolled population.

Months from Consent

Supplemental figure 2 – OS of the enrolled population.

Months from Consent

Supplemental figure 3 - Subgroup Analysis for PFS

Supplemental figure 4- Cumulative incidence of SM in the randomized population. (A) All SM in the whole population; (B) All according to LEN and OBS; (C) Solid tumors according to LEN and OBS; (D) Secondary Myelodysplastic Syndrome or Acute Myeloid Leukemia according to LEN or OBS.


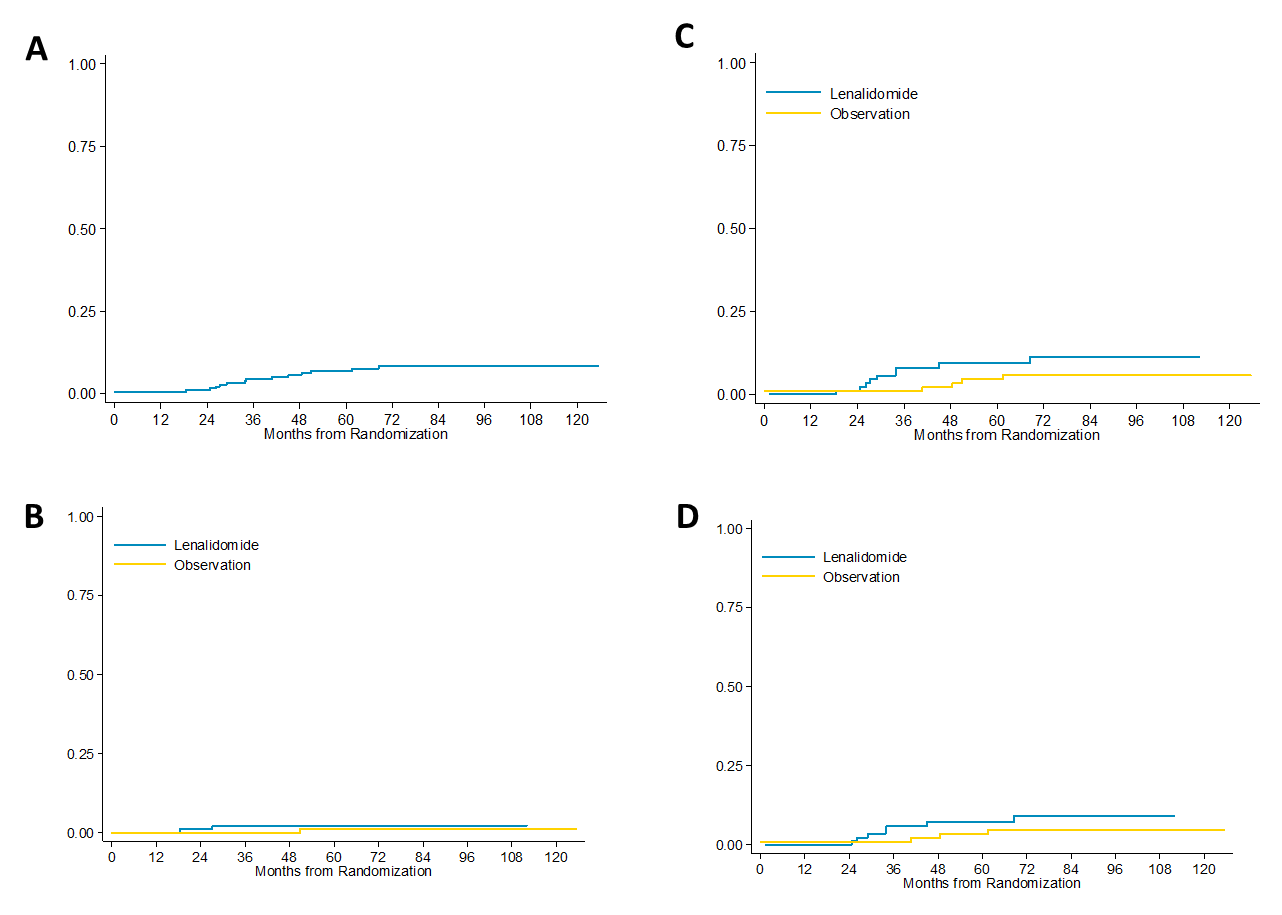

Supplemental figure 5 –MRD impact on overall survival, detected 6 months after ASCT. TTP stratified for MRD analyzed in BM (A) and PB (C). OS stratified for MRD analyzed in BM (B) and PB (C).

C

A

D

B

Supplemental figure 6- TTP stratified for MRD analyzed during the three years after ASCT in BM and PB, landmark analysis. A. MRD on BM and PD at M18 C-D. MRD on BM and PB at M24. E-F. MRD on BM and PB at M30. G-H. MRD on BM and PB at M36


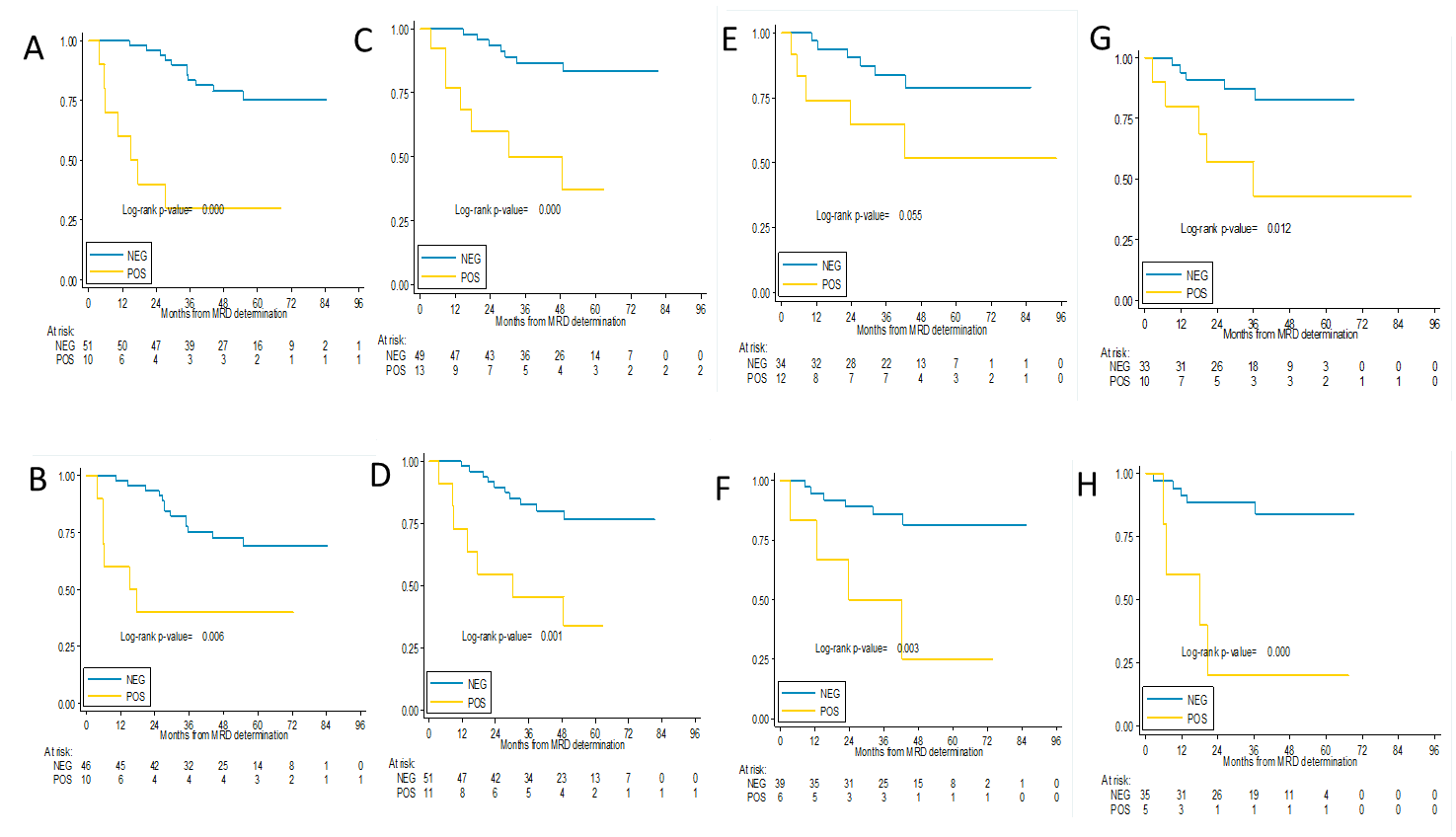


Supplemental figure 7 – Time-varying AUCs on MRD. A-B. Time-varying AUCs of TTP for BM and PB. C-D. Time-varying AUCs of OS for BM and PB.


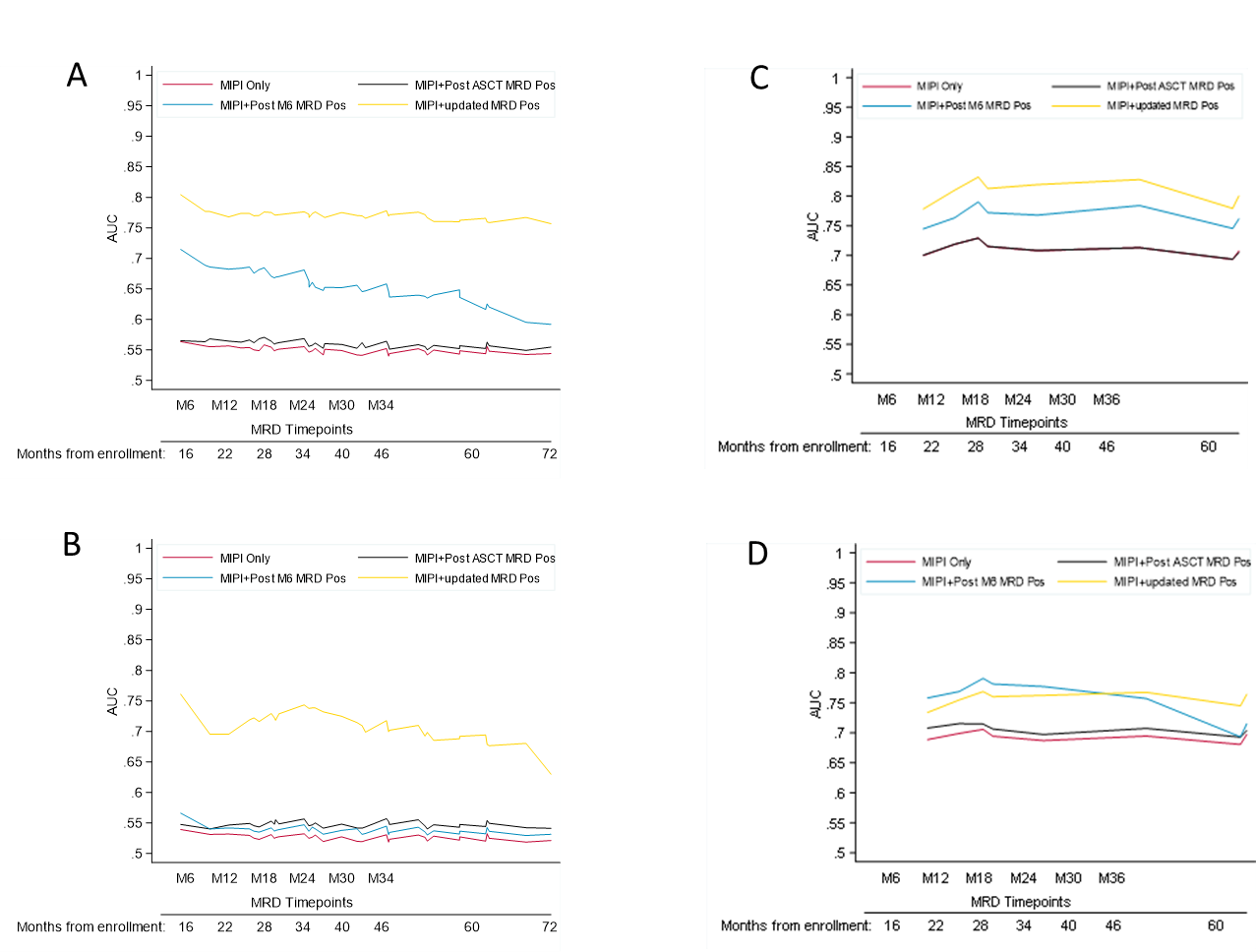

Supplement: Supplementary file 1 — Supporting information. [file HEM3-9-e70102-s001.docx]
